# Circulating Profiles of Serum Proguanylin, S100A12 Protein and Pentraxin 3 as Diagnostic Markers of Ulcerative Colitis

**DOI:** 10.3390/jcm12134339

**Published:** 2023-06-28

**Authors:** Aleksandra Kałużna, Agnieszka Jura-Półtorak, Alicja Derkacz, Julia Jaruszowiec, Krystyna Olczyk, Katarzyna Komosinska-Vassev

**Affiliations:** 1 Department of Clinical Chemistry and Laboratory Diagnostics, Faculty of Pharmaceutical Sciences in Sosnowiec, Medical University of Silesia in Katowice, 41-200 Sosnowiec, Poland; ajura@sum.edu.pl (A.J.-P.); julia.jaruszowiec@gmail.com (J.J.); olczyk@sum.edu.pl (K.O.); kvassev@sum.edu.pl (K.K.-V.); 2 City Hospitals of Chorzów, 41-500 Chorzów, Poland; derkacz.alicja@gmail.com

**Keywords:** proguanylin, S100A12, pentraxin 3, ulcerative colitis, inflammation, intestinal barrier integrity, neutrophils

## Abstract

The aim of this research was to investigate potential new biomarkers which could be used in the clinical practice of ulcerative colitis (UC). Given the crucial role of intestinal barrier integrity and inflammation in the pathogenesis of UC, the serum profile of proteins linked to intestinal barrier and pro-inflammatory neutrophil products may be useful in diagnosing and monitoring the activity of the disease. We measured serum levels of proguanylin (pro-GN), S100A12, and pentraxin 3 (PTX3) in 31 patients with UC before and after a year of biological treatment, as well as in 20 healthy individuals. Significant differences in the serum profiles of pro-GN (5.27 vs. 11.35, *p* < 0.001), S100A12 (39.36 vs. 19.74, *p* < 0.001) and PTX3 (3197.05 vs. 1608.37, *p* < 0.001) were observed between pre-treatment patients with UC and healthy individuals. Furthermore, in UC patients prior to treatment, the levels of S100A12 (*p* < 0.0005; r = 0.628) and PTX3 (*p* < 0.05; r = 0.371) were correlated with disease activity as measured by the Mayo scale. Following a year of biological treatment with adalimumab, the concentration of pro-GN significantly increased (5.27 vs. 6.68, *p* < 0.005) in the blood of UC patients, while the level of PTX-3 decreased (3197.05 vs. 1946.4, *p* < 0.0001). Our study demonstrates the usefulness of pro-GN, S100A12, and PTX3 measurements in diagnosing and monitoring the activity of UC.

## 1. Introduction

The prevalence of inflammatory bowel disease (IBD) is increasing globally and is anticipated to affect approximately 30 million people by 2025. IBD is a chronic disorder, characterized by inflammation in the intestinal tissue, which is believed to be the result of microbial dysbiosis, and environmental, genetic, and immunological factors. One of the primary types of IBD is ulcerative colitis (UC), which is characterized by inflammation of the intestinal mucosa that spread proximally from the rectum to other parts of the large intestine [1]. The diagnosis of UC is typically based on endoscopic examination, which is a valuable, albeit highly invasive diagnostic method. In cases of non-invasive assays, according to the European Crohn’s and Colitis Organisation (ECCO), measurements of fecal calprotectin (FC) are believed to be the most sensitive marker of intestinal inflammation in IBD. It should be noted, however, that FC is not specific only to IBD, as its concentration in feces may also be increased in other gastrointestinal disorders. Therefore, there is an urgent need to find new biomarkers which can support the diagnosis of UC and be used in monitoring disease activity. Developing new biomarkers may also provide additional insights into the underlying mechanism of IBD, which could ultimately improve disease management and patient outcomes [2,3,4].

The proper functioning of the intestines depends on various factors, including electrolyte homeostasis and the integrity of the intestinal barrier. The receptor guanylate cyclase C (GC-C), located on intestinal epithelial cells (IECs), plays a crucial role in both electrolyte homeostasis and intestinal barrier integrity. Guanylin, an endogenous GC-C agonist, is secreted into the intestinal lumen by IECs through auto- or paracrine mechanisms leading to the activation of GC-C. The precursor to guanylin is preproguanylin, which undergoes cleavage to form proguanylin (pro-GN), the most abundant inactive form of guanylin found in circulation. Under physiological conditions, the activation of GC-C by guanylin leads to the upregulation of intracellular cyclic guanosine monophosphate (cGMP), which, via the cystic fibrosis transmembrane conductance regulator (CFTR), induces the secretion of Cl^−^ and HCO_3_^−^ ions by the intestinal cells into the lumen [5,6]. An elevated level of cGMP inhibits the activity of the sodium–proton exchanger, resulting in a reduction of Na^+^ absorption by intestinal cells. The crucial role of GC-C signaling in the proper operation of the intestines is underscored by the fact that mutations of GC-C may lead to diarrhea or ileus [5]. GC-C signaling also plays a role in maintaining intestinal barrier integrity as it can regulate the proliferation, migration, and differentiation of IECs [7]. Moreover GC-C deficiency is believed to affect the expression of tight junction proteins (TJPs), including occludin, claudin-2, -4, and junctional adhesion molecules (JAMA), leading to impaired barrier integrity [6,8]. The disruption of intestinal barrier integrity is related to the enhanced penetration of intestinal cells by pathogenic antigens that can initiate or exacerbate immune response and the inflammatory process [1]. Given that the intestinal barrier plays a critical role in the pathogenesis of UC, as well as the importance of electrolyte homeostasis in the proper functioning of the intestines, assessments of GC-C activators may be useful in the diagnosis and management of UC. Therefore, pro-GN, which is the most abundant circulating precursor for GC-C agonists, is a promising candidate biomarker for UC.

Another of the new potential biomarkers of UC is S100A12, which, like calprotectin, belongs to the S100 protein family. The main cellular sources of S100A12 are neutrophils and to a lesser extent also monocytes, both of which play a role in the development of UC. Upon release from these cells, S100A12 can trigger a pro-inflammatory response by binding to the receptor for advanced glycation products (RAGE) and Toll-like receptor 4 (TLR-4). RAGE–ligand interactions result in the generation of reactive oxygen species (ROS), as well as the upregulation of the mitogen-activated protein kinase (MAPK) and nuclear factor kappa B (NF-κB) pathways, followed by the secretion of pro-inflammatory cytokines (TNF-α, IL-1β, IL-6, IL-8). The RAGE-mediated activation of NF-κB is characterized by prolonged persistence, which, when combined with the accumulation of RAGE ligands and the pathogenic role of ROS, may lead to enhanced inflammation and tissue damage. In addition, S100A12, via TLR-4, participates in monocyte activation exerting a significant role in the progression of UC [9,10,11]. Thus, the increased migration of neutrophils to the intestinal mucosa during UC may lead to the increased expression of S100A12 at the site of inflammation. At the same time, S100A12 contributes to the secretion of pro-inflammatory cytokines and monocyte migration, which leads to further release of S100A12 and a vicious cycle of inflammation [12]. Therefore, the identification of S100A12 as a potential biomarker for UC could be of great significance in the development of new diagnostic and therapeutic strategies for this disease.

Another biomarker investigated in our study is pentraxin 3 (PTX3), which is a glycoprotein that belongs to the pentraxin family, alongside C-reactive protein (CRP). Under physiological conditions, PTX3 remains at low levels, but it is rapidly synthesized and released by various cell types at sites of inflammation, particularly by neutrophils, dendritic cells (DC), monocytes, macrophages and fibroblasts in response to activation by Toll-like receptors (TLRs), agonists, and inflammatory cytokines such as IL-1β and TNF-α. PTX3 release may also occur as a result of the exposure of immune cells to microorganisms, in particular to lipopolysaccharide (LPS) or outer-membrane protein A (OmpA). Pentraxin 3 interacts with many ligands, such as extracellular matrix components, growth factors, and selected pathogens, and plays a role in complement activation, pathogen recognition by phagocytes, and the clearance of apoptotic cells [13,14,15,16,17,18]. During UC, the main cellular sources of PTX3 in gastrointestinal tissue are crypt-predominant neutrophils. Neutrophils serve as ready-to-use pentraxin 3 reservoirs, and can be released within minutes upon the activation of inflammation. Other innate cellular immune components, such as macrophages and dendritic cells, synthesize this molecule in response to pro-inflammatory signals. Upon the activation of inflammation, neutrophils release a small fraction of their PTX3 content. The rest of the secreted protein binds to the stem cell through neutrophil extracellular traps (NETs), which are abundant in inflamed sites. The release of PTX3, particularly by neutrophils, can elicit a cellular immune response in inflamed colon tissue, specifically purulent crypt lesions, in UC patients [13,16,17]. PTX3 also plays an important role in the tissue remodeling and repair processes of many diseases that lead to tissue damage. In response to the endogenous products of damaged cells and tissues, such as extracellular matrix components and nucleic acids, TLR and nucleotide-binding oligomerization domain-like receptors (NLRs) initiate inflammation. The production of inflammatory mediators and the recruitment of innate immune cells, including neutrophils or macrophages, further initiate tissue-repair processes. PTX3 produced by neutrophils, macrophages, and mesenchymal stem cells, interacts with the fibrin matrix and plasminogen, supporting pericellular fibrinolysis by tissue-remodeling cells, contributing to proper tissue repair. Studies have shown that PTX3 deficiency is associated with impaired pericellular fibrinolysis and the collective migration of remodeling cells, followed by an increase in fibrosis and damaged tissue repair [18]. Considering the above, PTX3 may be a useful new marker reflecting inflammation and tissue damage in autoimmune diseases, including patients with ulcerative colitis.

In light of the relationship of pro-GN with intestinal integrity, as well as with electrolyte homeostasis and its role in the pathogenesis of UC, pro-GN has emerged as a potential marker of UC. Excessive inflammatory response, the activation of immune cells, and the release of neutrophil-derived products such as S100A12 and PTX3 may occur as a result of the enhanced permeability of the intestinal barrier. Therefore, the aim of the study was to assess concentrations of pro-GN, S100A12 and PTX3 in the blood of patients with newly diagnosed ulcerative colitis and after one year of treatment with adalimumab. Additionally, the study aimed to investigate the effect of biological drug therapy on disease activity as measured on the Mayo scale, and the activity of ongoing inflammation, as reflected by the concentration of C-reactive protein. The study also assessed the relationships between serum levels of pro-GN, S100A12, PTX3, disease activity, and the intensity of inflammation.

## 2. Materials and Methods

### 2.1. Study Population

The biological material analyzed in the research included venous blood collected from 51 adults: 31 subjects with ulcerative colitis and 20 healthy controls. In the group of individuals with diagnosed UC, blood samples were collected both before the implemented treatment and a year after receiving biological treatment with adalimumab. The diagnosis of ulcerative colitis was made on the basis of endoscopic examination, laboratory tests and clinical symptoms. Additionally, disease activity was measured using the Mayo endoscopic scale and by the concentration of C-reactive protein, a systemic inflammation marker. The exclusion criteria for participation in this study among UC patients were unstable coronary disease, chronic kidney or liver disease, fulminant or toxic colitis, as well as pregnancy and breastfeeding. Patients receiving glucocorticoids treatment, live attenuated vaccines, ongoing rectal therapy with simultaneous therapeutic infusions, or previous biological treatment were also excluded. In the case of the control group, the exclusion criteria were hospitalization or surgery within the year, and pharmacological treatment during the previous month. Moreover, the values of morphological and biochemical analyses assessed in the blood of control subjects were required to fall within the reference ranges, as were BMI and blood-pressure values.

### 2.2. Assessing the Serum Proguanylin Concentration

Assessment of the serum proguanylin level was conducted using the Human Proguanylin ELISA Test from BioVendor Company (Karasek, Czech Republic). The analytical sensitivity in this test was estimated at the level of 0.06 ng/mL, while the intra-essay variability was 5.2%.

### 2.3. Assessing the Serum S100A12 Concentration

The measurements of S100A12 concentration in serum was performed using Human S100A12 ELISA test from BioVendor Company (Karasek, Czech Republic). The analytical sensitivity of used method was determined as 0.02 ng/mL and the intra-assay variability was 3.6%.

### 2.4. Assessing the Serum Pentraxin 3 Concentration

Levels of pentraxin 3 in the serum of patients with UC and healthy individuals were assessed using the Human Pentraxin 3 ELISA test from BioVendor Company (Karasek, Czech Republic). The sensitivity of the method was estimated as 22 pg/mL, while the intra-assay variability was 3.4%.

### 2.5. Statistical Analysis

The obtained data were evaluated using tSTATISTICA software (StatSoft 13.3, Kraków, Poland). Conducted The statistical analysis included verifying the normality of the data distribution using the Shapiro–Wilk test. The descriptive statistics of collected data consisted of: (1) median (Me); and (2) the interquartile range including lower (Q1) and upper (Q3) quartiles in cases of non-normally distributed data and mean values with a standard deviation (SD) in the normally distributed data. In order to verify the statistical significance of the differences between patients with UC and the control group, the Student’s *t*-test and Mann–Whitney U test were used in the case of normally and non-normally distributed data, respectively. To assess the impact of the implemented treatment on the investigated parameters, results were compared using the Student’s *t*-test and Wilcoxon test. The significance level was estimated as *p* < 0.05 for all tests and analyses performed in this research.

## 3. Results

### 3.1. Research Data

The clinical characteristic of patients with UC before (UC0) and after (UC1) treatment are summarized in Table 1. Data presented in Table 1 were published previously, as part of the research group was also included in a previous study [19]. It is interesting to note that in patients with UC before the implemented treatment, median disease activity was evaluated as having 3 points according to the Mayo endoscopic score. After a year of biological treatment, the median disease activity decreased by 1 point in the Mayo score and was statistically significant. The median CRP level before and after treatment remained low and did not exceed the reference rage defined as lower than 10 mg/L. The CRP levels within the reference rage were noted in the case of 21 (68%) and 25 (81%) patients with UC before and after treatment, respectively. The CRP level indicating severe colitis was defined according to ECCO recommendations [2] as being higher than 30 mg/L, and such a situation was noted in the case of six (19%) patients in the pre-treatment group and one (3%) patient in the post-treatment group. The results of circulating levels of serum proguanylin (pro-GN), S100A12 protein, and pentraxin 3 (PTX-3) in the study individuals, control subjects, patients with UC before the implemented of biological treatment, and after a year of therapy with adalimumab, are presented in Table 2.

### 3.2. Serum Level of Pro-GN, S100A12 and PTX3 in Patients with Ulcerative Colitis and Healthy Individuals

**Figure 1:**
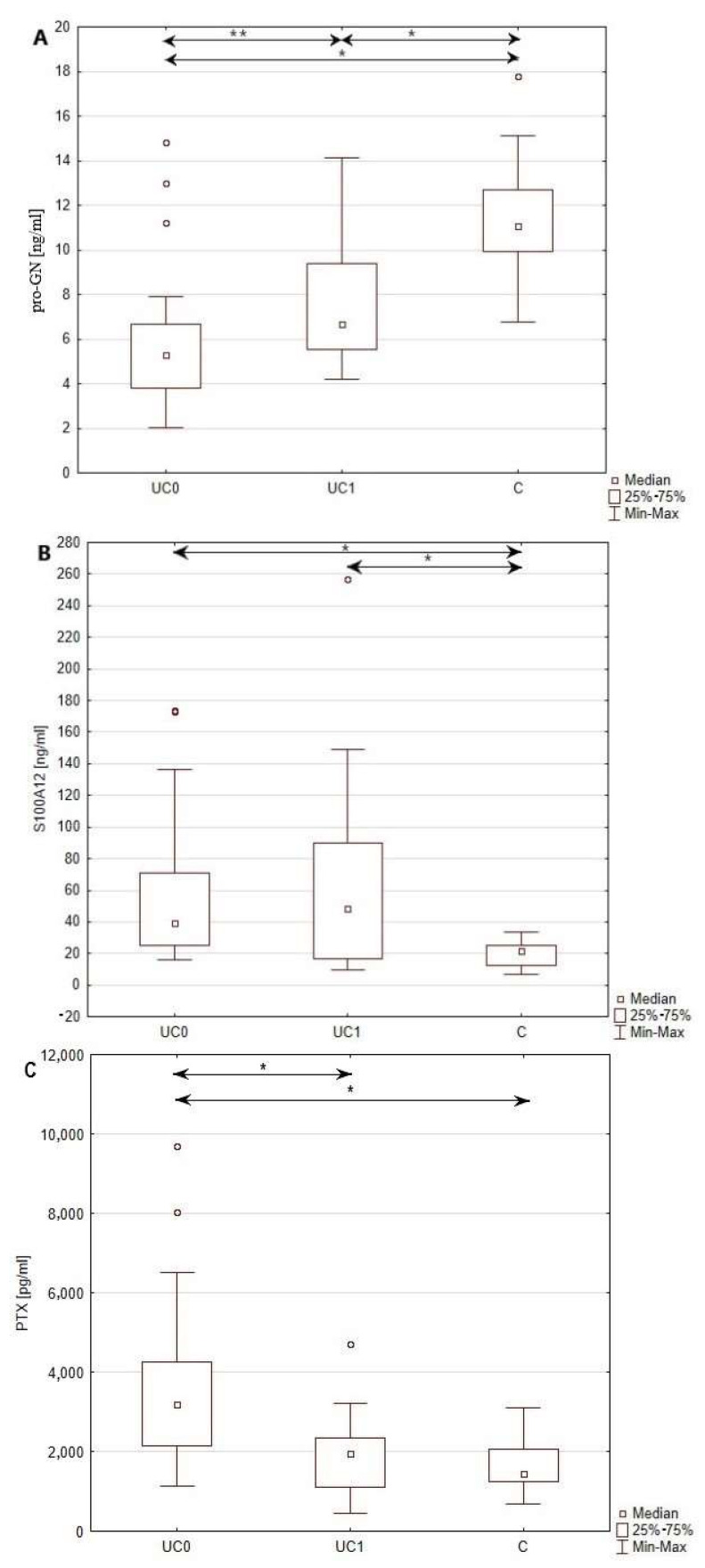
Comparison of serum profiles of pro-GN (**A**), S100A12 (**B**) and PTX3 (**C**) in the group of patients with UC before, as well as after, a year of Adalimumab therapy, and in healthy individuals *—*p* < 0.001; **—*p* < 0.005; C—healthy individuals; UC0—patients with UC before biological treatment; UC1—patients with UC after a year of treatment.

In the pre-treatment group of patients with UC, as well as after a year of biological therapy serum, concentrations of pro-GN were significantly decreased (*p* < 0.0001) compared to healthy individuals. The observed decrease of the pro-GN level reached 46.4 and 58.9% pre- and post-treatment. Concentrations of S100A12 (*p* < 0.0001) were, in contrast to pro-GN, significantly increased compared to the healthy individuals. Observed increase of S100A12 level reached 99.5% in the group of patients before treatment, and 144.9% in the group after treatment. In the case of PTX3 (*p* < 0.0001), significant difference between healthy individuals and patients with UC was noted only prior to treatment implementation. Patients with UC who qualified for biological treatment presented a significant change (98.8%) in PTX3 levels compared to the control group. The serum profiles of pro-GN, S100A12 and PTX3 in patients with UC, both before and after treatment, as well as those of the control group, are illustrated in the Figure 1.

### 3.3. The Relationship between Serum Pro-GN, S100A12, PTX3 and Inflammatory Process and Disease Activity

The relationship between the serum concentrations of pro-GN, S100A12 and PTX-3 and the presence of ongoing inflammation in intestinal tissues as assessed by measuring C-reactive protein was evaluated. The statistical analysis revealed a significant correlation between S100A12 and CRP concentrations after treatment (r = 0.48, *p* < 0.05); however, no similar correlation was found between these parameters before treatment. In the case of PTX3 and pro-GN, the correlations between their serum levels and CRP levels in patients with UC were not significant both before and after treatment with adalimumab. Post-treatment patients with UC presented a lower level of CRP; however, the difference was not statistically significant.

**Figure 2:**
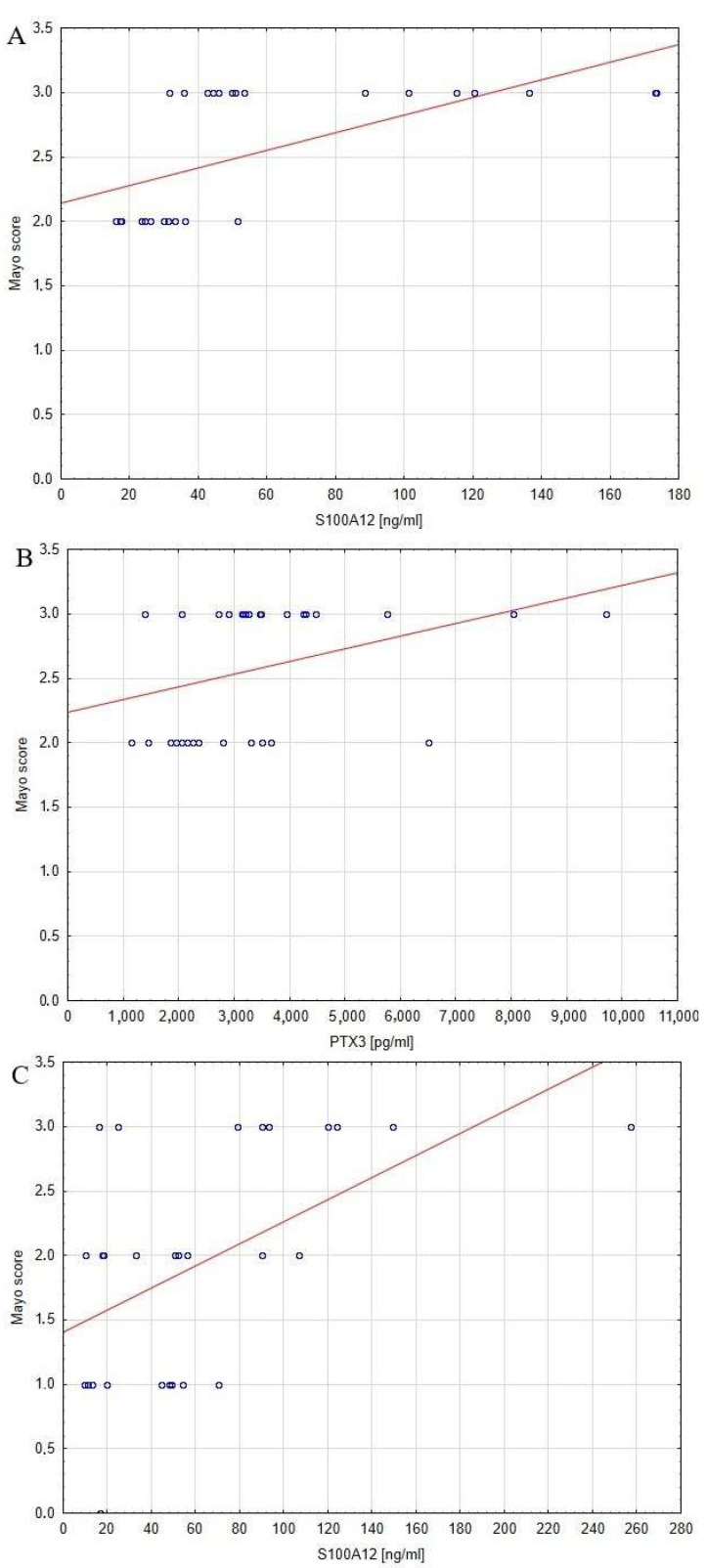
Significant relationships between the S100A12, PTX3 concentrations, and disease activity expressed in the Mayo score in patients with UC before and after biological treatment: (**A**) correlation between S100A12 level and disease activity before treatment; (**B**) correlation between PTX3 level and disease activity before treatment; and (**C**) correlation between S100A12 level and disease activity after treatment; blue circles—function from biomarker serum concentration to Mayo score; red line—regression line.

The relationship between serum profiles of pro-GN, S100A12, PTX3 and disease activity was also evaluated. The significance of correlations between serum profile of analyzed biomarkers and markers of inflammation and disease activity are presented in Table 3. Statistically significant relationships between concentrations of the analyzed biomarkers and disease activity are presented as graphs in Figure 2. The results of our study showed that the serum concentration of pro-GN did not correlate with disease activity. No such correlation was found in patients with UC, either before or after, adalimumab therapy. On the other hand, the statistical analysis revealed a significant correlation between concentrations of S100A12 and the Mayo scale before (r = 0.6, *p* < 0.0005) and after (r = 0.5, *p* < 0.005) adalimumab treatment. Moreover, the abovementioned relationship further confirmed as patients with 2 points on the Mayo scale in the group before treatment showed lower serum concentrations (*p* < 0.05) of S100A12 compared to those who obtained 3 points of disease activity on the Mayo scale. In the case of PTX3, noted a statistically significant relationship between the level of the analyzed protein and the Mayo scale in the pre-treatment (r = 0.4, *p* < 0.05) group of patients with UC. After year of adalimumab treatment, disease activity decreased by an average of 1 point on the Mayo scale (*p* < 0.0005), demonstrating the beneficial effect of the implemented therapy.

### 3.4. The Influence of One Year of Biological Treatment with Adalimumab on the Serum Profile of Pro-GN, S100A12 and PTX3

Implemented biological treatment with adalimumab affect the concentrations of serum pro-GN (*p* < 0.005) and PTX3 (*p* < 0.0001), however no significant difference was noted in case of S100A12. Serum pro-GN level increased by 26.8% after a year of biological treatment and was still significantly different compared to healthy individuals. In contrast to pro-GN concentration of serum PTX3 in group of patients with UC decreased after a year of treatment by 60.9%, reaching the values characteristic for healthy individuals.

## 4. Discussion

### 4.1. Serum Profile of the Pro-GN, S100A12 and PTX3 in Patients Diagnosed with UC and Healthy Individuals

The pathogenesis of ulcerative colitis is related to disrupted intestinal barrier integrity, gut dysbiosis, and a reduction in the mucus that protects the intestines. These factors contribute to the penetration of IECs by microbial and dietary antigens. It remains unclear whether the impaired integrity of the intestinal barrier is a trigger or a consequence of the pathological process, but it certainly plays a crucial role in the progression of UC [9,10]. Given the critical role of barrier integrity in disease progression, we assessed the concentrations of pro-GN—the precursor of a GC-C agonist in the serum of patients with UC. A significant difference in the pro-GN levels of UC patients and healthy individuals was found in our study. Pro-GN concentrations in the serum of patients with UC before, as well as after, treatment was decreased compared to the control group. Unfortunately, there are no studies evaluating the concentrations of pro-GN in the serum of patients with UC; however, Lan et al. [20] noted that the expression of active forms of pro-GN was significantly reduced in the colonic biopsies of patients with UC. The observations made by Lan et al., and those found in our study, suggest that the downregulation of pro-GN and guanylin expression may result in the downregulation of GC-C signaling, leading to a hydro-electrolyte imbalance in the intestines. Precise concentrations of Cl^−^, Na^+^, HCO_3_^−^ ions are crucial to maintaining the rheological properties of the mucus that protects the intestinal mucosa. Moreover, decreased GC-C signaling may compromise the intestinal barrier’s integrity, as it regulates the proliferation and differentiation of IECs [7,20]. In a mouse model study, Lin et al. [8] noted that GC-C deficiency led to the reduced expression of TJPs such as occludin, claudin-2, -4, and JAMA, and also caused intestinal hyperpermeability to macromolecules. A reduced level of pro-GN may lead, by lowering the level of GC-C, to increased intestinal barrier permeability, mucus dysfunction, and imbalances in the water and electrolytes in the intestines. Given the potential role of pro-GN in the initiation and progression of the disease, as well as the abovementioned findings, serum levels of pro-GN appear to be a valuable diagnostic marker for UC.

Disruption of the intestinal barrier integrity observed over the course of UC may increase the exposure of IECs to pathogenic antigens, which in turn activates the immune response and the inflammatory process through the secretion of pro-inflammatory cytokines, including IL-13, TNF-α, IFN-γ [21,22,23]. An important role in the initiation and progression of inflammation is also played by neutrophil-derived products exhibiting pro-inflammatory properties, such as S100/A12 [9,10]. In our study we assessed the concentrations of this protein in the serum of pre- and post-treatment patients with UC and found higher levels of S100A12 in both groups of patients with UC compared to healthy individuals. The alterations observed in our study may have potentially arisen from the augmented secretion of the protein from neutrophils that infiltrate intestinal tissue during the inflammatory process. The abovementioned neutrophil-derived products may further percolate into systemic circulation, which results in their increased concentration in the serum of UC patients. Neutrophil activation results in the degranulation of their granules, leading to the release of various molecules, including myeloperoxidase, neutrophil elastase, and S100A12. The latter protein actively contributes to the promotion of inflammation through its binding RAGE, which subsequently activates the pro-inflammatory MAPK and NF-κB pathways. This process further enhances inflammatory response and elevates the release of S100A12 from neutrophils and/or macrophages, thereby instigating a vicious cycle of inflammation [9,10,24]. Increased serum levels of S100A12 in patients with UC was also reported by Foell et al. [25], thus confirming the results of our research. Evaluations of S100A12 concentrations in the serum of patients with UC have been limited compared to those made in fecal studies [26]. However, measurements of S100A12 in both biological materials could serve as a valuable diagnostic tool for UC.

Similar to S100A12 proteins, one of the main sources of PTX3 during UC are intestinal neutrophils [13]. Thus, enhanced activation of neutrophils during UC may result in the excessive release of not only S100A12, but also PTX3. Our study showed a significant rise in the serum levels of PTX3 among patients with UC in contrast to healthy individuals. The observed elevated level of PTX3 may also have been the result of an increased level of pro-inflammatory cytokines such as TNF-α, which may have contributed to the upregulation of PTX3 among the patients with UC. Our findings align with Kato et al.’s research [17], which highlighted a significant increase in concentrations of pentraxin 3 in the plasma of patients with UC compared to healthy individuals [27]. Therefore, measurements of circulating PTX3 concentrations could be employed as a novel diagnostic biomarker for UC.

### 4.2. The Relationship between Disease Activity and Serum Profile of Pro-GN, S100A12 and PTX3 in Patients with UC

In this study we assessed the relationship between disease activity, as measured by the Mayo endoscopic scale, and the serum profiles of pro-GN, S100A12 and PTX3 in patients with UC. No significant relationship was found between levels of pro-GN and disease activity. However, a significant correlation was observed between the Mayo scale and S100A12 values, both before and after treatment. Moreover, patients with a higher score of disease activity had significantly increased plasma concentrations of S100A12. The obtained results are in line with the previous study by Foell et al. [25], which reported higher serum S100A12 concentrations among patients with active disease compared to those with inactive disease. The primary source of the observed high levels of S100A12 in the UC is likely to have been neutrophils, which are the predominant components of intestinal infiltrates. Secreted S100A12 can bind to the RAGE receptor, the expression of which is unsignificant, under physiological conditions, on monocytes or other cells. However, the activation of transcription factors or the accumulation of ligands such as S100A12, leads to the induction of RAGE expression in the inflammation associated with UC. S100A12 may act as a chemoattractant for neutrophils and macrophages, which may result in further releases of S100A12. Consequently, an accumulation of S100A12 in the intestinal tissue may lead to the upregulation of NF-κB by activating RAGE. The NF-κB pathway regulates the expression of not only cytokines and adhesion molecules, but also RAGE, which may provoke further activation of NF-κB and the progression of inflammation, including increased the mobilization and migration of immune cells (such as neutrophils) to lesions. Thus, the abovementioned viscous cycle of inflammation may lead to elevated serum levels of S100A12 among patients with active disease [11,28]. As a result, the serum profile of S100A12 can be useful in monitoring disease activity, thereby reducing the need for more invasive assays. In the case of other neutrophil-derived products exhibiting pro-inflammatory properties such as PTX3, we found a significant relationship to the Mayo score prior to treatment, while after treatment the relationship between the variables was not statistically significant. These results are consistent with those of Kato et al. [17], who reported increased plasma PTX3 levels in patients with active disease compared to those in clinical remission, and a significant correlationship between PTX3 levels and the Mayo score. Moreover, Ashour et al. [27] demonstrated that PTX3 levels in colonic tissue were elevated in a mouse model of colitis compared to mice without colitis. These researchers suggested that pentraxin 3 levels rapidly increased in response to an inflammatory process, with these levels being higher after two weeks of colitis induction than levels observed after four weeks of induction. The observed correlation between PTX3 and UC disease activity highlights the potential role of this glycoprotein in monitoring UC activity.

### 4.3. The Influence of Biological Treament with Adalimumab on the Circulating Profile of Pro-GN, S100A12 and PTX3

The effects of the adalimumab treatment on the circulating levels of pro-GN, S100A12 and PTX3 were assessed in this study. After one year of biological treatment, a significant difference in pro-GN concentrations was observed in the serum of patients with UC. This increase in the level of pro-GN after anti-TNF treatment may be attributed to the relationship between analyzed protein and TNF-α, as previously noted by Brenna et al. [29]. In this study, pro-GN expression in the intestinal biopsies of patients with IBD was negatively correlated with the expression of TNF-α. Furthermore, in a study using the HT-29-18-N2 cells as a goblet cell-like culture model, Harmel-Laws et al. [30] found that exposure to TNF-α led to a decrease in guanylin expression. These observations suggest that TNF-α is a significant suppressor of guanylin expression and that anti-TNF-α treatment may increase the levels of both guanylin and its precursor, proguanylin. Based on these observations, it can be inferred that serum pro-GN assessments may be used to determine the effectiveness of the anti-TNF-α biological treatment in reducing TNF-α expression.

Despite improvement in disease activity after one year of adalimumab treatment in patients with UC, no significant difference in the concentration of serum S100A12 was noted. The lack of a significant difference in S100A12 concentrations in the serum may have been due to the fact that neutrophil and monocyte counts did not change after adalimumab treatment, as these cells are the main sources of the abovementioned protein. Unfortunately, no studies to date have evaluated the effect of anti-TNFα treatment on the serum levels of S100A12 in patients with UC. However, Boschetti et al. [31] conducted a similar study on a group of patients with another type of IBD, Crohn’s disease. After 14 weeks of anti-TNF treatment, the researchers found a significant decrease in the concentrations of S100A12 in the serum of patients with Crohn’s disease. It is noteworthy that S100A12 is expressed constitutively by neutrophils, but its expression by monocytes can be induced by TNF-α, LPS or IL-6 [32,33]. Thus, while the implemented anti-TNF treatment with adalimumab may suppress one inducer of S100A12 expression, it does not affect the influence of LPS and IL-6 on S100A12 levels.

In addition to S100A12, PTX3 is another pro-inflammatory product derived from neutrophils that was analyzed in our study. The obtained results revealed that the implemented therapy with a biological drug resulted in a statistically significant decrease in PTX3 concentrations in the serum of patients with UC after one year of treatment. This finding suggests that anti-TNF-α treatment not only ameliorates inflammation throughout the intestines, but also potentially contributes to a decrease in PTX3 levels, as TNF-α stimulates the release of this glycoprotein. Considering the notable difference in PTX3 concentrations after treatment implementation and the lack of a similar difference in the case of other proteins belonging to the pentraxin family such as CRP, PTX3 measurements appear to be better markers of inflammatory processes and treatment response in UC, compared to CRP.

## 5. Conclusions

A significant difference in the serum profiles of pro-GN, S100A12 and PTX3 between patients with UC and healthy individuals was observed in our study. These biomarkers have potential utility in the diagnosis of UC. Notably, a significant correlation between serum S100A12 levels and the Mayo score was observed in UC patients before and after treatment, suggesting that S100A12 may be used as a marker of disease activity. Moreover, since serum pro-GN and PTX3 levels differed significantly after the implemented treatment, they may be utilized as markers to evaluate responses to treatment. In contrast, no significant difference was noted in the case of CRP, indicating that pro-GN and PTX3 seem to better reflect response to treatment than CRP, which is a more common inflammatory marker. Our study provides new insights into the diagnosis and management of ulcerative colitis, which can be further explored and possibly introduced in clinical practices.

## Figures and Tables

**Table 1:** Clinical characteristics of patients with UC before the implemented treatment (UC0) and after a year of receiving biological treatment with adalimumab (UC1).

Parameter	UC0	UC1	*p*
Mayo score	3 (2–3)	2 (1–3)	**0.000**
CRP [mg/L]	3.4 (1.26–17.51)	2.47 (1.51–7.68)	0.160
Glucose [mmol/L]	4.99 ± 0.70	4.81 ± 0.81	0.293
Cholesterol [mmol/L]	4.98 ± 0.79	4.93 ± 0.91)	0.724
Triglycerides [mmol/L]	1.23 (1.01–1.49)	1.0 (0.87–1.36)	**0.017**
Indirect bilirubin [μmol/L]	4.75 (1.80–7.70)	8.3 (5.50–16.00)	**0.000**
Direct bilirubin [μmol/L]	3.45 (1.90–3.80)	5.30 (3.50–8.20)	**0.000**
ALT [U/L]	15.00 (10.00–26.00)	14.50 (10.00–23.00)	0.591
AST [U/L]	17.92 ± 4.81	19.42 ± 6.18	0.136
Creatnine [μmol/L]	77.8 (68.50–87.90)	74.70 (63.40–87.10)	0.117
Total protein [g/L]	73.48 ± 5.43	74.73 ± 5.63	0.231
Albumin [g/L]	42.52 ± 4.73	43.34 ± 4.52	0.358
Sodium [mmol/L]	140 (138.00–142.00)	140.00 (138.00–141.00)	0.381
Potassium [mmol/L]	4.17 ± 0.40	3.97 ± 0.33	**0.011**
Calcium [mmol/L]	2.36 ± 0.09	2.33 ± 0.12	0.660
Hemoglobin [g/dl]	12.83 ± 2.28	13.49 ± 2.29	**0.005**
Neutrophils [%]	66.65 ± 11.25	64.52 ± 10.92	0.282
Lymphocytes [%]	24.27 ± 10.71	26.99 ± 11.74	0.259
Basophils [%]	0.76 ± 0.43	0.71 ± 0.37	1.000
Eosinophils [%]	2.6 (1.10–3.30)	2.03 ± 1.43	0.063
Monocytes [%]	5.72 ± 2.27	5.75 ± 1.79	0.859
PLT [×10^9^/L]	375.93 ± 108.79	342.04 ± 101.72	**0.043**

Comparison of selected biochemical and morphological parameters assessed in the blood of patients with UC, before and after a year of treatment with adalimumab. Results are presented as mean ± standard deviation in normally distributed data, and median and interquartile range in not-normally distributed data. In all the conducted statistical tests, a significance level of *p* < 0.05 was used. Data with statistical significance are bolded. ALT, alanine aminotransferase; AST, aspartate aminotransferase; CRP, C-reactive protein; PLT, platelets count.

**Table 2:** Characteristics of serum concentrations of proguanylin, S100A12 protein, and pentraxin 3 concentrations in the serum of pre- and post-treatment patients with UC and healthy individuals.

Parameter	UC0	UC1	C
Pro-GN [ng/mL]	5.27 (3.80–6.65) #	6.68 (5.51–9.36) #	11.35 ± 2.59 *
S100A12 [ng/mL]	39.36 (25.15–70.99) #	48.32 (16.84–89.99) #	19.74 ± 8.07 *
PTX3 [pg/mL]	3197.05 (2148.20–4248.95) #	1946.4 (1103.61–2334.93) #	1608.37 ± 587.05 *

Results are presented as mean ± standard deviation [*] in normally distributed data and median with interquartile range [#] in not-normally distributed data. PTX3, pentraxin 3; Pro-GN—proguanylin.

**Table 3:** The significance of correlation between serum profile of analyzed proteins disease activity.

**Parameter**		**Mayo Score**
Pro-GN	UC0	*p* > 0.05
	UC1	*p* > 0.05
S100A12	UC0	***p*** ** < 0.0005**
	UC1	***p*** ** < 0.005**
PTX3	UC0	***p*** ** < 0.05**
	UC1	*p* > 0.05

Data with statistical significance has been bolded.

## Data Availability

Data are included within the article.
